# Evaluating 3D-Patch Efficacy in Wound Healing Using the Medicinal Leech *Hirudo verbana* as an In Vivo Model

**DOI:** 10.3390/nano16120712

**Published:** 2026-06-09

**Authors:** Giorgia Costantini, Laura Pulze, Nicolò Baranzini, Elisabetta Campodoni, Monica Sandri, Annalisa Grimaldi

**Affiliations:** 1 Department of Biotechnology and Life Sciences (DBSV), University of Insubria, Via J.H. Dunant 3, 21100 Varese, Italy; gcostantini@uninsubria.it (G.C.); laura.pulze@uninsubria.it (L.P.); nicolo.baranzini@uninsubria.it (N.B.); 2 Institute of Science, Technology and Sustainability for Ceramics (ISSMC), National Research Council (CNR), Via Granarolo 64, 48018 Faenza, Italy; elisabetta.campodoni@issmc.cnr.it (E.C.); monica.sandri@issmc.cnr.it (M.S.)

**Keywords:** leech, wound healing, 3D patch, magnesium-doped hydroxyapatite

## Abstract

Skin injuries are common and can result from surgeries, burns, pressure sores, cuts, and diseases. Proper wound healing is crucial for maintaining homeostasis; wounds can be classified as acute or chronic. Acute wounds heal in four sequential phases: hemostasis, inflammation, proliferation, and remodeling. Chronic wounds arise when this process fails, often due to prolonged inflammation. Existing treatments for chronic wounds are limited, and antibiotic resistance complicates infection control, highlighting the urgent need for new therapies. Biomaterials, particularly gelatin, have gained attention for their biomimetic properties, biocompatibility, and ability to promote healing. Gelatin’s ECM-like structure supports tissue metabolism, and it can be enriched with bioactive compounds to enhance tissue regeneration, collagen deposition, angiogenesis, and antimicrobial activity. This study evaluates the effectiveness of a 3D gelatin-based patch in vivo, using *Hirudo verbana* as a model. The patch, functionalized with chitosan and bioactive apatite nanoparticles, was implanted in injured leeches, with tissue samples collected at 72 h, 1 week, and 2 weeks. Scaffold integration, cell colonization, and healing effects were assessed through morphological, immunohistochemical, and ultrastructural analyses. The findings confirm *H. verbana* as a robust in vivo model for regenerative medicine and demonstrate the promising potential of gelatin-based patches.

## 1. Introduction

Skin injuries, ranging from acute surgical wounds to debilitating chronic ulcers, represent a significant clinical challenge. While acute wounds typically follow a structured healing process, hemostasis, inflammation, proliferation, and remodeling, chronic wounds remain stalled in a pro-inflammatory state, necessitating the development of advanced bioactive scaffolds [1,2,3,4,5,6]. Traditional treatments frequently fail to address the complex microenvironment of non-healing wounds.

In this context, tissue engineering has shifted toward 3D porous scaffolds that mimic the extracellular matrix (ECM) [7,8]. Among natural polymers, gelatin and chitosan are widely used due to their biocompatibility and ability to promote cell adhesion [9,10,11,12]. To enhance these scaffolds, the integration of inorganic phases like hydroxyapatite (HA) has shown great promise [13,14,15]. Specifically, magnesium-doped hydroxyapatite (MgHA) allows for the incorporation of foreign ions and their controlled release, while magnesium ions play a crucial role in promoting cell migration and adhesion [15,16,17].

In our previous work, we characterized a novel composite 3D patch (GelMgHA@GelChit) and demonstrated its initial biocompatibility and integration in an invertebrate model [17]. Building on those findings, the current study aims to provide a comprehensive biological characterization of the regenerative processes triggered by this material. We utilize the medicinal leech, *H. verbana*, an established and sophisticated model for studying innate immunity and tissue repair. Unlike vertebrates, the leech possesses a simplified but highly conserved system of wound healing, characterized by a rapid and stereotyped response involving specific cell populations [18,19,20,21,22,23].

This study investigates the recruitment and activation of myoendothelial precursors and myofibroblasts during the healing process. By monitoring markers such as CD31, CD34, MyoD, and fibroblast growth factor receptors (FGFr), we aim to elucidate how the GelMgHA@GelChit patch modulates the cellular microenvironment to favor organized tissue reconstruction. This research highlights the efficacy of the leech model in evaluating advanced biomaterials, adhering to the 3Rs principle by providing a valid alternative to vertebrate testing.

## 2. Materials and Methods

### 2.1. Patch Development and Characterization

The gelatin–chitosan-based patch, functionalized with nano-hydroxyapatite hybrid particles (GelMgHA), GelMgHA@GelChit was prepared according to a previously reported procedure [17]. Briefly, 10 mL of a 12 wt% gelatin (Gel) aqueous solution was prepared by dissolving the polymer in water at 40 °C. Subsequently, 20 mL of chitosan (Chit) solution (2 wt%), obtained by dissolving 0.4 g of chitosan powder in a 1% (*v*/*v*) aqueous acetic acid solution, was added to the gelatin, obtaining the Gel-Chit blend. The resulting mixture was mechanically stirred at 37 °C for 30 min. In parallel, an aqueous suspension of GelMgHA hybrid particles (0.97 g in 25 mL) was prepared and sonicated for 10 min using a tip sonicator (VCX130, Sonics & Materials, Newtown, CT, USA) in an ice bath to ensure proper dispersion and to reduce particle aggregation. The Gel-Chit blend was then added to the GelMgHA suspension at 37 °C and maintained under magnetic stirring overnight to achieve complete homogenization, yielding a composite hydrogel (GelMgHA@GelChit).

The composite was subsequently processed into patches by freeze-drying using a controlled cycle: freezing at a rate of −50 °C/h down to −40 °C, followed by controlled heating at 5 °C/h from −40 °C to −5 °C, and then at 3 °C/h up to 20 °C. The entire process lasted approximately 3 days under vacuum (P = 0.086 mbar). Finally, the obtained dried patches were subjected to dehydrothermal (DHT) treatment at 120 °C for 48 h under a pressure of 0.01 mbar to enhance scaffold stability through the formation of covalent crosslinks within the polymeric network.

The patch morphology and the pore size were observed by environmental scanning electron microscopy (SEM TM Quanta 200, FEI, Thermo Fisher Scientific Inc., Waltham, MA, USA), set in High Vacuum (P < 10^−4^ Torr) mode. The samples were fixed on aluminium stubs using carbon tape, and they were coated with Au using coating units Polaron Sputter Coater E5100 (Polaron Equipment, Watford, Hertfordshire, UK).

Inductively Coupled Plasma–Optical Emission Spectrometry (ICP-OES, Agilent Technologies 5100, Santa Clara, CA, USA) was conducted using a Liberty 200 spectrometer (Varian, Palo Alto, CA, USA) for the quantitative determination of Mg^2+^, Ca^2+^, and PO_4_^3−^ ions, which constitute the inorganic mineral component. In brief, 20 mg of hybrid patch samples were dissolved in 50 mL of a 2 wt% HNO_3_ solution prior to the analysis. XRD patterns were obtained using a D8 Advance diffractometer (Bruker, Karlsruhe, Germany) equipped with a Lynx-eye position-sensitive detector. The analysis employed Cu Kα radiation (λ = 1.54178 Å) at 40 kV and 40 mA. Spectra were recorded in the 2θ range from 20° to 80 °C, with a step size (2θ) of 0.02° and a counting time of 0.5 s. The thermal properties of the samples were assessed using a STA 449 F3 Jupiter instrument (Netzsch, Geraetebau, Germany), also used to quantify the amount of MgHA mineral phase nucleated on gelatine molecules (80 wt%) and the total content incorporated in the patches (30 wt%). Simultaneous thermal gravimetric analysis (TGA) and differential scanning calorimetry (DSC) were conducted in alumina crucibles, from room temperature to 1100 °C, at a heating rate of 10 °C/min under a nitrogen flow. The sample weighed approximately 10 mg.

To assess the fluid uptake capacity [17], cylindrical samples of the patch (diameter: 6 mm, height: 0.2–2 mm) were immersed in a PBS solution at 37 °C until saturation was reached. At specified intervals (0.5, 1, 2, 4, 6, 24, 48 h), samples were removed; excess water was removed using filter paper and then weighed. The equilibrium swelling ratio was determined using the formula:

Swelling ratio = (wet weight − dry weight)/(wet weight) × 100

The measure was repeated in triplicate on different samples.

For degradation assessments [17], cylindrical patch samples (diameter: 6 mm, height: 0.2–2 mm) were placed in phosphate-buffered saline (PBS, pH 7.2) with 0.1% (wt/vol) NaN3 at 37 °C. In the case of patches obtained through the solvent casting technique, an initial freeze-drying step was performed to eliminate any residual water from the samples. At specific time intervals (1, 3, 7, and 14 days), samples were retrieved, washed twice with Milli-Q water, freeze-dried for two days, and then reweighed. Degradation percentage (D (%)) was calculated using the equation:

D (%) = (Wi − Wf)/Wi × 100

where Wi is the initial weight of the freeze-dried sample before immersion in PBS, and Wf is the weight of the freeze-dried sample at a specific time point.

The moisture permeability of the prepared patch was determined following the ASTM E96/E96M; Standard Test Methods for Gravimetric Determination of Water Vapor Transmission of Materials [24]. Initially, a vial (diameter = 1.50 cm) containing 10 mL of MilliQ water was covered with the patch as a lid and weighed (W0). An open vial and a vial sealed with its lid served as positive and negative controls, respectively. After placement in a humidity chamber at 37 °C and 50% relative humidity for 24, 48, and 72 h, they were reweighed (Wt). Water Vapor Transmission Rate (WVTR) of the patch was computed with the formula:

WVTR (g m^−2^ d^−1^) = ((Wt − W0)/t)/A

where A is the exposed area of the patch (m^−2^), and t is the time of measurement (day). The values were expressed as the mean ± standard error (*n* = 3).

### 2.2. Animal Treatment

Leeches (*Hirudo verbana*, Anellida, Hirudinea) measuring about 10 cm were kindly donated by Italian Leech Farm (ILFARM s.r.l., Varese, Italy) and kept in aerated tanks at 20 °C in lightly salted water (NaCl 1.5 g/L in distilled water). Before each experiment, leeches were anesthetized with 10% ethanol solution. For the implantation, at about the 30th and the 80th superficial metamere from the oral sucker, leeches were surgically injured with a razor. Immediately, patches measuring 2 mm × 2 mm × 2 mm, previously cut and re-hydrated in PBS buffer, were placed into the wound. To avoid patch loss due to contraction of the muscular body wall, grafts were sutured with Optilene^®^ surgical synthetic monofilament (B. Braun: Melsungen, Germany) and then a drop of liquid band aid was applied upon the wound. A control group was also established, in which leeches received a 5 mm body wall incision with a razor [19] but no scaffold implantation, to monitor the physiological healing process. Grafted animals were then kept in moist chambers for a recovery period of 2 h and subsequently placed in water tanks. At specific time points (72 h, 1 w, 2 w), stitches were removed, and the leech body tissues containing the patch were dissected and processed for the experimental uses reported in the following paragraphs. In these experiments, three animals were randomly assigned to each of the experimental and control groups using a computer-generated randomization list (Microsoft Excel, Microsoft Corp., Redmond, WA, USA). Each animal was assigned a unique identifier, and allocation to groups was performed by matching these identifiers to the randomization list to ensure an unbiased and equal distribution across groups. Each group was composed of three different animals, and for each animal, experiments were conducted in triplicate. This sample size (*n* = 3) and the use of technical triplicates follow our established laboratory protocols for statistical analysis, as consistently applied in our previous studies [25,26].

### 2.3. Morphological Analysis at Light Microscopy, Transmission (TEM) and Scanning (SEM) Electron Microscopy

The portions of the leech containing the grafted patches were removed and fixed with 4% glutaraldehyde in 0.1 M cacodylate buffer (pH 7.4) for 2–4 h and then washed three times for 10 min in the same buffer. Then, tissues were post-fixed in 1% osmium tetroxide (OsO4) for 2 h in the dark and then washed in the cacodylate buffer. Subsequently, samples were dehydrated with increasing concentrations of ethanol (50%, 70%, 90%, 100%) and embedded in an Epon-Araldite 812 mixture (Sigma Aldrich, Milan, Italy). Sections for light microscopy (0.7 μm in thickness) were obtained with a Reichert Ultracut S ultratome (Leica, Wein, Austria), stained by the conventional methods with crystal violet and basic fuchsine and observed under the light microscope. For TEM analysis, from the same samples, ultrathin sections (70 nm in thickness) were obtained with a Reichert Ultracut S ultratome (Leica), placed on copper grids (400 mesh, Sigma Aldrich, Milan, Italy), counterstained by uranyl acetate and lead citrate, and observed with a JEOL1400Plus transmission electron microscope (Centro di Ricerca e Trasferimento Tecnologico [CRIETT], University of Insubria, Varese, Italy). Data were recorded with a MORADA digital camera system (Olympus, Tokyo, Japan).

For SEM analysis, sections (12 μm) of the specimens embedded in paraffin were obtained with a rotatory microtome (Jung multicut 2045, Leica Microsystems, Nussloch, Germany) and collected with gelatin-coated slides. Sections were rehydrated and washed in PBS for 5 min. Then slices were post-fixed in a solution of 0.1% osmium tetroxide in PBS overnight. Finally, sections were dehydrated in an increasing series of ethanol (70%, 80%, 95% and 100%, 5 min each) and two times (5 min each) in hexamethyldisilazane (Sigma Aldrich, Milan, Italy). Dried slices were mounted on carbonated stubs, gold-coated in an Emitech K250 sputter coater (Emitech, Baltimore, MD, USA) and observed with a 360 Zeiss Gemini 360 (Centro di Ricerca e Trasferimento Tecnologico [CRIETT], University of Insubria).

### 2.4. Masson’s Trichrome Staining

Tissues have been fixed in 4% paraformaldehyde for 2 h and then washed three times in PBS buffer. Subsequently, samples were dehydrated in an increasing scale of ethanol (30%, 50%, 70%, 90%, 96%, and 100%) and paraffin-embedded. Sections (7 μm) were obtained with a rotatory microtome (Jung multicut 2045, Leica), collected with gelatin-coated slides and processed for Masson trichrome staining (ready-to-use kit, Bio Optica, Milan, Italy). Samples were observed under a light microscope (Eclipse, Nikon, Tokyo, Japan).

### 2.5. Indirect Immunofluorescence Staining

Cryosections (7 μm) were obtained with a cryotome (Leica CM1850, Wetzlar, Germany) and collected on gelatin-coated slides. For indirect immunofluorescence, cryosections were rehydrated for 5 min with PBS (pH 7.4) and then pre-incubated for 30 min with BSA blocking solution (2% Bovine Serum Albumin, 0.1% Tween20 diluted in PBS; the same solution was also used to dilute all the antibodies). Samples were then incubated for 90 min with the primary antibodies (Table 1) and, after washes with PBS, were incubated for 1 h with the secondary antibody conjugated with cyanin 3 (Cy3, ThermoFisher Scientific, dilution 1:500). After several washes with PBS, nuclei were counterstained with 4′,6-Diamidino-2-phenylindole (DAPI 0.1 mg/mL in PBS) for 7 min and the slides were mounted with Citifluor (Citifluor Ltd., London, UK). Negative control experiments were performed by omitting primary antibodies. Slides were finally observed under a fluorescence microscope (Eclipse Nikon) equipped with the emission filters 360/420 nm for DAPI nuclear staining and 550/580 nm for CY3. The images obtained were combined using Adobe Photoshop (Adobe Systems, Inc., San Jose, CA, USA).

### 2.6. Quantitative Image Analysis and Statistical Methods

Fluorescent signal intensities for FGFr, CD31, CD34, and MyoD were evaluated by analyzing 10 random fields (45,000 μm^2^ each) per experimental group using the ImageJ software package (ImageJ software package (https://imagej.net/software/imagej/, accessed on 1 June 2026, National Institutes of Health, Bethesda, MD, USA, version 1.53e). To quantify the signal, we measured the Mean Fluorescence intensity area after applying a consistent thresholding method. Specifically, background subtraction was performed using a rolling ball radius of 50 pixels, and images were converted to 8-bit format. Thresholding was applied using the Default algorithm to distinguish positive signal from background noise; this threshold remained constant across all images within the same experimental set to ensure objective comparison. All image acquisitions and quantifications were performed blindly to minimize observer bias. Statistical analyses were performed using Prism GraphPad 8 (GraphPad Software, Boston, MA, USA). Data normality and homoscedasticity were verified via Shapiro-Wilk and Levene tests, respectively. Differences between time points were assessed using a one-way ANOVA followed by Tukey’s post hoc test. Differences were considered statistically significant at *p* < 0.05. Data in graphs are presented as averages ± standard deviation (SD), and asterisks indicate statistically significant differences between the analyzed time points.

## 3. Results

### 3.1. Patch

**Figure 1:**
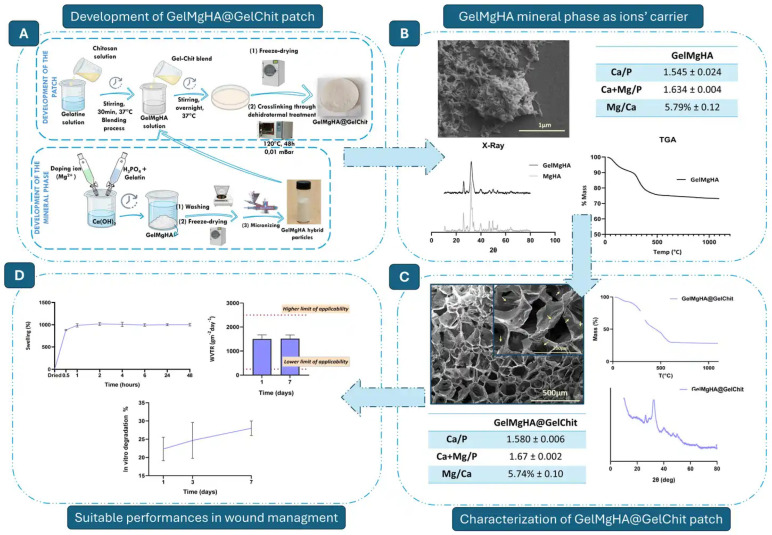
Schematic overview of the development process, characterization, and functional performance of the GelMgHA@GelChit patch. (**A**) Description of (i) the synthesis process, via neutralization process, of GelMgHA hybrid particles, (ii) their incorporation into a gelatin–chitosan matrix and iii) fabrication procedure to obtain the GelMgHA@GelChit porous patch. (**B**) Characterization of GelMgHA hybrid particles, including morphology and compositional analysis by ICP, XRD and TGA, highlighting the formation of low-crystalline Mg-doped hydroxyapatite. (**C**) Morphological and physicochemical characterization of the GelMgHA@GelChit patch, showing a highly porous and interconnected structure, with a MgHA/polymer wt% ratio of 30/70, with the preservation of MgHA characteristics. The arrows highlight GelMgHA crystals embedded within the matrix. (**D**) Evaluation of functional properties relevant to wound healing, including swelling behaviour, in vitro degradation, and water vapor transmission rate (WVTR), demonstrating suitable performance for wound management applications. Adapted from Ref. [17] under a CC-BY 4.

The GelMgHA@GelChit patch was developed as a bioresorbable hybrid matrix combining antimicrobial capability with intrinsic bioactivity for tissue regeneration. A key component of this system is represented by GelMgHA hybrid nanoparticles, obtained through a biomimetic mineralization process in which gelatin acts as an organic template for apatite nucleation (Figure 1A). The interaction between Ca^2+^ ions and the carboxylic groups of gelatin promotes the formation of nanostructured apatite directly on the polymer chains, while the presence of Mg^2+^ ions leads to the formation of a poorly crystalline magnesium-doped hydroxyapatite closely resembling natural apatite. The resulting particles consist of needle-like mineral aggregates organized around the gelatin matrix, forming micrometric flakes. The partial substitution of Ca^2+^ with Mg^2+^ and the presence of the organic phase promote the formation of a low-crystalline and highly bioresorbable mineral phase (Figure 1B) [17,27,28].

These biohybrid particles were embedded within a gelatin–chitosan (3:1 *w*/*w*) polymeric network at a GelMgHA/polymer weight ratio of 30/70, leading to the formation of the GelMgHA@GelChit patch (Figure 1C). The freeze-drying process generated a highly porous three-dimensional structure characterized by an open and interconnected network with pore sizes in the order of hundreds of micrometers and macroporosity exceeding 80% (Figure 1C). This architecture is particularly advantageous for regenerative applications, as it facilitates cell adhesion, infiltration, and nutrient transport. At higher magnification, the GelMgHA particles appeared homogeneously distributed throughout the matrix and contributed to the surface roughness of both pore walls and external surfaces, without altering their intrinsic chemical properties [28,29]. The mineral phase (Figure 1C) retained its characteristic low-crystalline GelMgHA nature after processing, as shown by XRD, confirming the stability of the hybrid particles within the polymeric scaffold [26]. Furthermore, the GelMgHA@GelChit patch exhibited a mineral content of approximately 30 wt%, ensuring a significant bioactive contribution within the matrix. The polymeric network, composed of gelatin and chitosan, provides a combination of bioactivity and antimicrobial properties, with gelatin promoting cell adhesion and chitosan contributing to structural integrity and antibacterial effects [17].

The highly porous structure of the GelMgHA@GelChit patch resulted in rapid fluid uptake and high swelling capacity (up to ~1100%), indicating an effective ability to absorb exudates and maintain a moist environment. However, this pronounced swelling is accompanied by a relatively fast degradation, as evidenced by significant mass loss within the first 24 h and a progressive transition towards a softer hydrogel-like state over time. Despite this, the patch exhibited water vapor transmission rate values within the optimal range (~1500 g/m^2^/day), ensuring proper moisture balance. Overall, these results highlight a trade-off between high fluid handling capacity and limited structural stability, while still confirming the suitability of the system for wound healing applications requiring efficient exudate management (Figure 1D) [17].

The developed patch combines high porosity and adequate vapor permeability, both essential for maintaining an optimal wound environment. Its interconnected structure supports effective exudate absorption while promoting cell adhesion, infiltration, and nutrient diffusion, and also enables straightforward drug loading through absorption.

The incorporation of bioresorbable GelMgHA hybrid particles likely enhances the scaffold’s biological activity. Although Mg^2+^ release was not directly quantified in this study, the observed regenerative response may be partly attributed to the sustained release of Mg^2+^ ions, which are known to promote cell migration, matrix remodeling, and wound healing [30,31]. Regarding mechanical performance, the GelMgHA@GelChit patch is designed as a soft, bioresorbable dressing rather than a load-bearing scaffold; thus, its stability was evaluated via swelling, WVTR, degradation, and in vivo integration. Future investigations will include mechanical testing (tensile/compressive) to further characterize its handling properties under clinically relevant conditions.

Collectively, these features highlight the potential of the patch as a multifunctional platform for advanced wound healing applications.

### 3.2. Time-Course of Patch Colonization and Tissue Remodeling: From Histology to Ultrastructure

The integration of the patch into the leech tissue and the subsequent cellular colonization were evaluated through a combined chronological analysis using light and electron microscopy.

**Figure 2:**
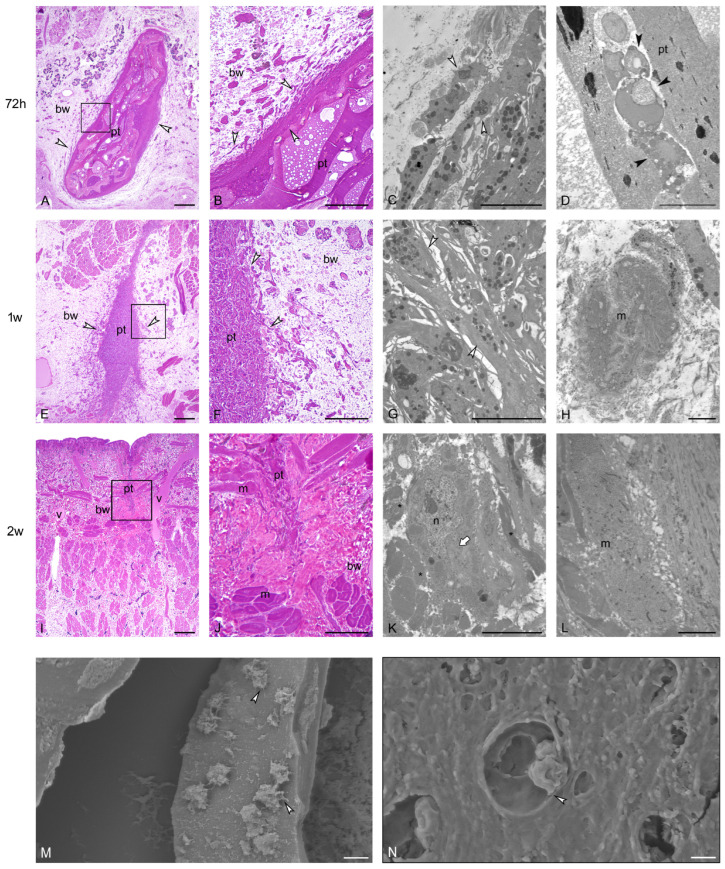
Histological and ultrastructural characterization of the implanted patch. (**A**,**B**) Light microscopy images at 72 h post-implantation showing the patch (pt) integrated into the host body wall (bw). A dense population of spindle-shaped cells (white arrowheads) is visible surrounding the graft, forming a thin pseudoblastema. (**C**) TEM analysis identifies these as vasocentral cells (white arrowheads), featuring electron-dense cytoplasm and large secretory granules. (**D**) Detail of individual cells (black arrowhead) infiltrating the internal mesh fibers of the patch (pt). (**E**,**F**) At 1 week, the patch structure (pt) appears heavily remodeled and colonized by vasocentral cells (white arrowheads), as further confirmed by TEM in (**G**). (**H**) TEM micrograph showing the differentiation of early muscle fibers (m) along the graft edge. (**I**,**J**) By 2 weeks, the implant site is nearly indistinguishable from the surrounding body wall (bw) mainly composed of muscle cells (m); newly formed blood vessels (v) are evident, facilitating the recruitment of myogenic precursors. (**K**) High-magnification TEM of a muscle precursor cell, showing a prominent nucleus (n), nascent myosin filaments (arrow), and adjacent bundles of newly synthesized collagen (asterisks). (**L**) TEM image of a maturing muscle fiber (m) with organized contractile elements. (**M**,**N**) SEM micrographs at 72 h and 1 week, respectively, illustrating the robust adhesion and spreading of colonizing cells (white arrowheads) onto the patch fibers. Black boxes in (**A**,**E**,**I**) indicate the magnified regions shown in (**B**,**F**,**J**), respectively. Scale bars: (**A**,**E**,**I**): 100 μm; (**B**,**F**,**J**): 50 μm; (**G**): 10 μm; (**C**,**D**,**K**): 5 μm; (**H**,**L**–**N**): 2 μm.

At 72 h, the patch was already integrated into the host tissue, although its structure and meshes remained clearly visible (Figure 2A,B). The graft was surrounded by numerous spindle-shaped cells that infiltrated even the innermost parts, establishing a thin pseudoblastema. TEM analysis confirmed the presence of these cells between the patch meshes (Figure 2D) and identified them as vasocentral cells, characterized by an electron-dense cytoplasm containing a few large granules (Figure 2C). In accordance with our previous findings [17,32], these cells spontaneously differentiate into myofibroblasts to facilitate wound closure and initial remodeling of the surrounding loose connective tissue.

By one week, the physical structure of the patch was no longer recognizable under light microscopy due to complete colonization by vasocentral cells (Figure 2E,F), which continued to migrate actively toward the implant site (Figure 2G). TEM imaging at this stage revealed the onset of myogenesis, with newly differentiating muscle fibers clearly identifiable within the extracellular matrix surrounding the patch (Figure 2H).

After two weeks, the implant was almost indistinguishable from the host tissue (Figure 2I,J). The muscle tissue appeared largely regenerated, with well-organized muscle fiber bundles occupying the sub-marginal wound area. TEM observations further elucidated this recovery, showing myocytes with prominent nuclei and cytoplasm rich in myosin filaments, alongside nascent muscle fibers (neo-fibers) where contractile material was systematically organizing into distinct sarcomeres. Notably, this stage was characterized by the presence of newly formed blood vessels and myoendothelial precursor cells (Figure 2K), which are instrumental in recruiting additional muscle precursors to the site. While a reduced pseudoblastema remained near the implant, it was encased in a significantly more compact and remodeled connective tissue.

To better characterize the physical interaction between the host cells and the synthetic structure, SEM analysis was performed. These observations allowed for a detailed assessment of the interface between cells and patch, demonstrating the high affinity of the colonizing cells for the material and their effective adhesion to the patch meshes (Figure 2M,N).

### 3.3. Masson’s Tricrome Staining

To evaluate the dynamics of collagen deposition and extracellular matrix (ECM) remodeling, Masson’s Trichrome staining was performed at three consecutive time points, comparing the patch-treated group with a control group undergoing natural wound healing. As shown in the Supplementary Data (Figure S1), the control group exhibited a typical physiological repair process characterized by a standard inflammatory infiltrate and a less organized connective tissue deposition. The direct comparison between the two groups highlights significant differences in the regenerative pace and quality, which are clear by the varying intensity of the blue staining.

**Figure 3:**
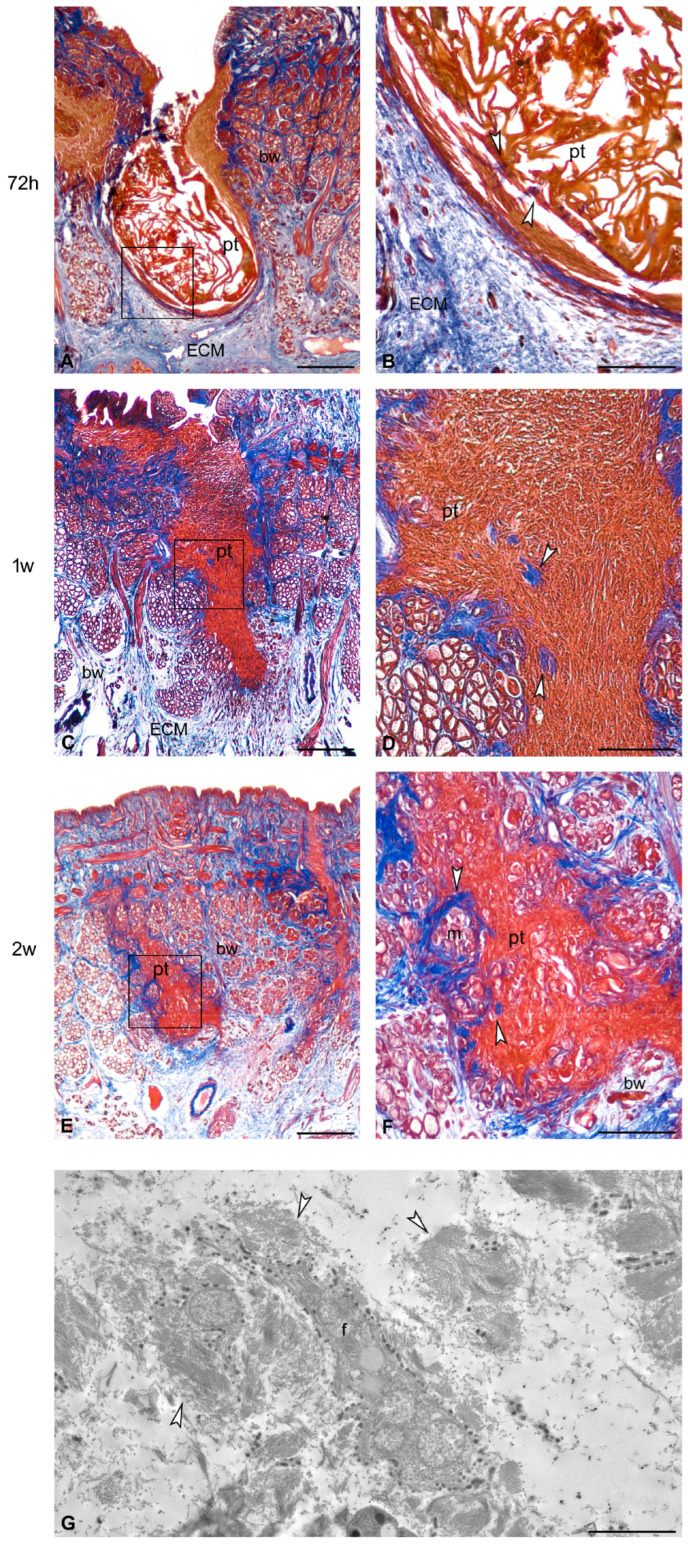
Histological and ultrastructural analysis of collagen deposition. (**A**,**B**) Masson’s Trichrome staining at 72 h post-implantation shows the patch (pt) surrounded by a loose extracellular matrix (ECM) within the host body wall (bw). Initial collagen fibers (white arrowheads) are sparse and primarily localized along the peripheral edges of the graft. (**C**,**D**) At 1 week, a significant increase in collagen density (white arrowheads) is observed, with blue-stained fibers encapsulating the implant. Sparse collagen deposits are also detectable within the inner regions of the patch (pt). (**E**,**F**) By 2 weeks, localized areas of dense, mature collagen provide structural support to newly formed muscle fibers (m) infiltrating the graft site. (**G**) TEM micrograph reveals fibroblast-like cells (f) characterized by an active secretory phenotype, observed in the process of synthesizing and depositing collagen fibrils (white arrowheads) into the surrounding space. In Masson’s Trichrome staining, collagen fibers are identified by their blue coloration. Scale bars: (**A**,**C**,**E**): 250 μm; (**B**,**D**,**F**): 100 μm; (**G**): 2 μm.

At 72 h post-injury, the control group (Figure S1) displayed significantly pale and diffuse blue staining, typical of a standard healing process where collagen fibers are still sparse and poorly organized. In contrast, the grafted patch was already surrounded by a light-blue-stained matrix (Figure 3A,B). While this indicates an initial phase of the regenerative response, a higher concentration of fibers was already detectable along the inner margins of the scaffold compared to the control.

By one week, the differences became more pronounced. In the natural healing group (Figure S1), the connective tissue remained relatively disorganized and less dense. Conversely, the patch-treated samples (Figure 3C) appeared completely colonized and were encased in a dense, dark-blue-stained connective tissue, signaling the formation of a more compact and mature collagenous matrix. Notably, the marked increase in blue color intensity within the scaffold meshes (Figure 3D) suggests active localized deposition and robust ECM remodeling facilitated by the synthetic structure.

Two weeks post-operation, the superior structural consolidation in the patch-treated tissue was evidenced by an intense and well-distributed blue staining (Figure 3E,F), providing a stable scaffold for the newly organizing muscle fibers. At the same stage, the control group (Figure S1) still exhibited a significantly less organized and sparser collagenous framework. These observations were further supported by TEM analysis (Figure 3G), which identified activated myofibroblasts actively synthesizing and depositing nascent collagen fibers into the surrounding microenvironment, confirming the patch’s role in promoting a more efficient and structured regenerative process.

### 3.4. Immunofluorescence Characterization of Myofibroblast Activation and Patch Infiltration

**Figure 4:**
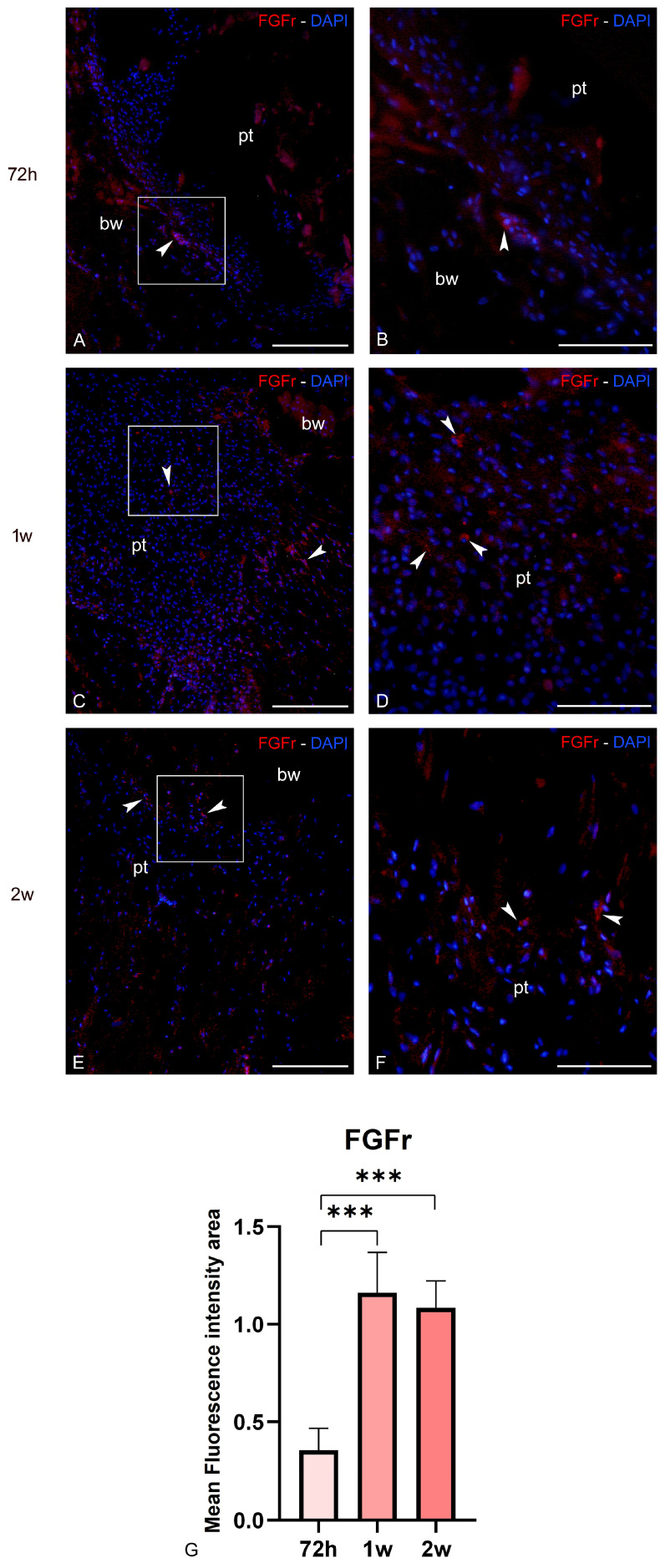
Spatiotemporal distribution of FGFr^+^ cells during patch colonization and remodeling. (**A**,**B**) Immunofluorescence images at 72 h post-implantation showing FGFr^+^ cells (red; white arrowheads) primarily localized at the periphery of the patch (pt), marking the interface with the host body wall (bw). Nuclei are counterstained with DAPI (blue). (**C**,**D**) At 1 week and (**E**,**F**) 2 weeks post-implantation, the FGFr^+^ signal (white arrowheads) becomes progressively more widespread. Immunopositive cells are clearly detectable both surrounding the patch (pt) and deeply infiltrating the inner mesh regions. (**G**) Quantitative analysis of the fluorescence intensity area confirms a significant increase in FGFr expression at 1 week. Statistical significance is indicated as *** (*p* < 0.001). (*p* < 0.001), which persists at high levels through the second week. White boxes in (**A**,**C**,**E**) indicate the magnified regions shown in (**B**,**D**,**F**), respectively. Scale bars: (**A**,**C**,**E**): 100 μm; (**B**,**D**,**F**): 50 μm.

To further characterize the cellular populations infiltrating the patch, immunofluorescence assays were performed using an anti-FGFr antibody (Figure 4). This receptor, in vertebrates, is a well-known marker for fibroblast-like cells differentiating into myofibroblasts, the key cellular components responsible for collagen production and tissue reorganization during healing [33,34]. FGFr was also employed in the leech model to identify the activated myofibroblasts. These cells, organized into the pseudoblastema, represent the primary functional units driving the structural remodeling of the tissue surrounding and infiltrating the implant [32,35].

At 72 h post-implantation (Figure 4A,B), FGFr-positive (FGFr+) cells were primarily detected at the host-tissue/patch interface. This initial recruitment marks the early assembly of the vasocentral-derived pseudoblastema around the patch, where these activated elements begin to orchestrate the healing response. By 1 week (Figure 4C,D) and 2 weeks (Figure 4E,F), a high density of FGFr+ cells was observed both surrounding and deeply infiltrating the patch. This progressive inward migration confirms that the activated population effectively colonizes the synthetic mesh, driving the synthesis of the new extracellular matrix and ensuring the overall integration of the graft into the body wall. Quantitative analysis of FGFr immunofluorescence showed a significant increase in fluorescence intensity from 72 h to 1 week, with a sustained high signal at 2 weeks, confirming the progressive recruitment and persistence of activated myofibroblast-like cells during patch remodeling (Figure 4G).

### 3.5. Progressive Cell Infiltration and Marker Expression Within the Patch

To further characterize the precursor populations infiltrating the patch, immunofluorescence microscopy was employed using CD31, CD34 and MyoD antibodies. According to the literature on the leech model [19,21], these three markers are essential for identifying myoendothelial precursor cells, a versatile population capable of contributing to both vascular and muscular regeneration.

**Figure 5:**
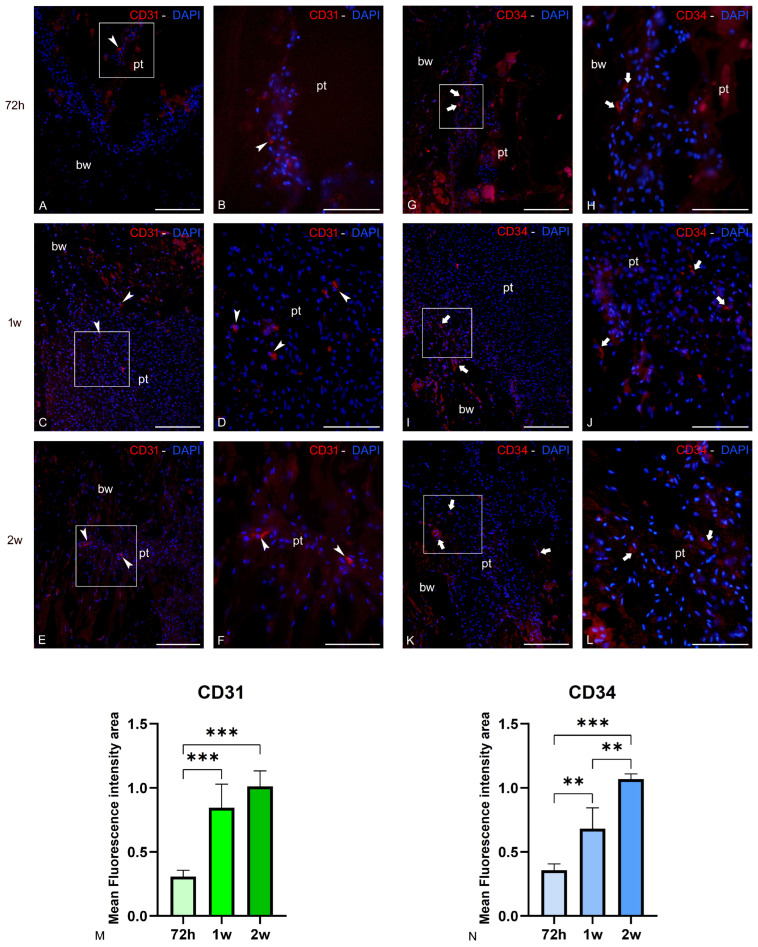
Recruitment of CD31^+^ and CD34^+^ endothelial and progenitor cells. (**A**–**F**) Representative immunofluorescence images of CD31 expression (red) and (**G**–**L**) CD34 expression (red) at 72 h, 1 week, and 2 weeks post-implantation. Nuclei are counterstained with DAPI (blue). (**A**,**B**,**G**,**H**) At 72 h, CD31^+^ and CD34^+^ cells are primarily confined to the interface between the host body wall (bw) and the patch (pt). (**C**,**D**,**I**,**J**) By 1 week and (**E**,**F**,**K**,**L**) 2 weeks, both markers show a more widespread distribution, indicating progressive infiltration of host cells into the inner regions of the graft and successful tissue integration. Arrowheads (CD31) and arrows (CD34) point to representative immunoreactive cells. (**M**,**N**) Quantitative analysis of the fluorescence intensity area for both markers confirms a significant and progressive increase in signal over time (*p* < 0.01; Statistical significance is indicated as *** (*p* < 0.001). White boxes in (**A**,**C**,**E**,**G**,**I**,**K**) indicate the magnified regions shown in (**B**,**D**,**F**,**H**,**J**,**L**), respectively. Scale bars: (**A**,**C**,**E**,**G**,**I**,**K**): 100 μm; (**B**,**D**,**F**,**H**,**J**,**L**): 50 μm in the figure.

Immunofluorescence analysis of CD31 and CD34 (Figure 5A–L) revealed a progressive colonization of the patch by positive cells over time. At 72 h, CD31 (Figure 5A,B) and CD34 (Figure 5G,H) immunoreactive cells were mainly localized at the interface between the patch and the surrounding tissue, with only a limited number of positive cells detectable within the biomaterial matrix. This early distribution suggested the initial recruitment of host-derived cells from the adjacent tissue into the patch.

At 1 week, the number and distribution of CD31 (Figure 5C,D) and CD34 (Figure 5I,J) positive cells increased markedly. Immunoreactive cells were observed not only at the periphery but also in deeper regions of the patch, indicating a more extensive infiltration of the implant by cells with an endothelial-associated phenotype. The broader spatial distribution of these cells suggested that the patch supported active tissue integration and the establishment of a permissive microenvironment for vascular-related remodeling.

At 2 weeks, CD31 (Figure 5E,F) and CD34 (Figure 5K,L) immunoreactivity remained detectable throughout the patch and at the host–patch interface. Positive cells appeared more numerous and more widely dispersed than at earlier time points, consistent with ongoing cellular infiltration and progressive remodeling of the implanted material. Overall, the temporal pattern of CD31/CD34 expression indicated that the patch facilitated host cell ingress and the maintenance of a vascular-associated regenerative response. Quantitative analysis confirmed these observations, showing a significant increase in CD31 and CD34 fluorescence intensity from 72 h to 1 week, with sustained elevated levels at 2 weeks (Figure 5M,N).

**Figure 6:**
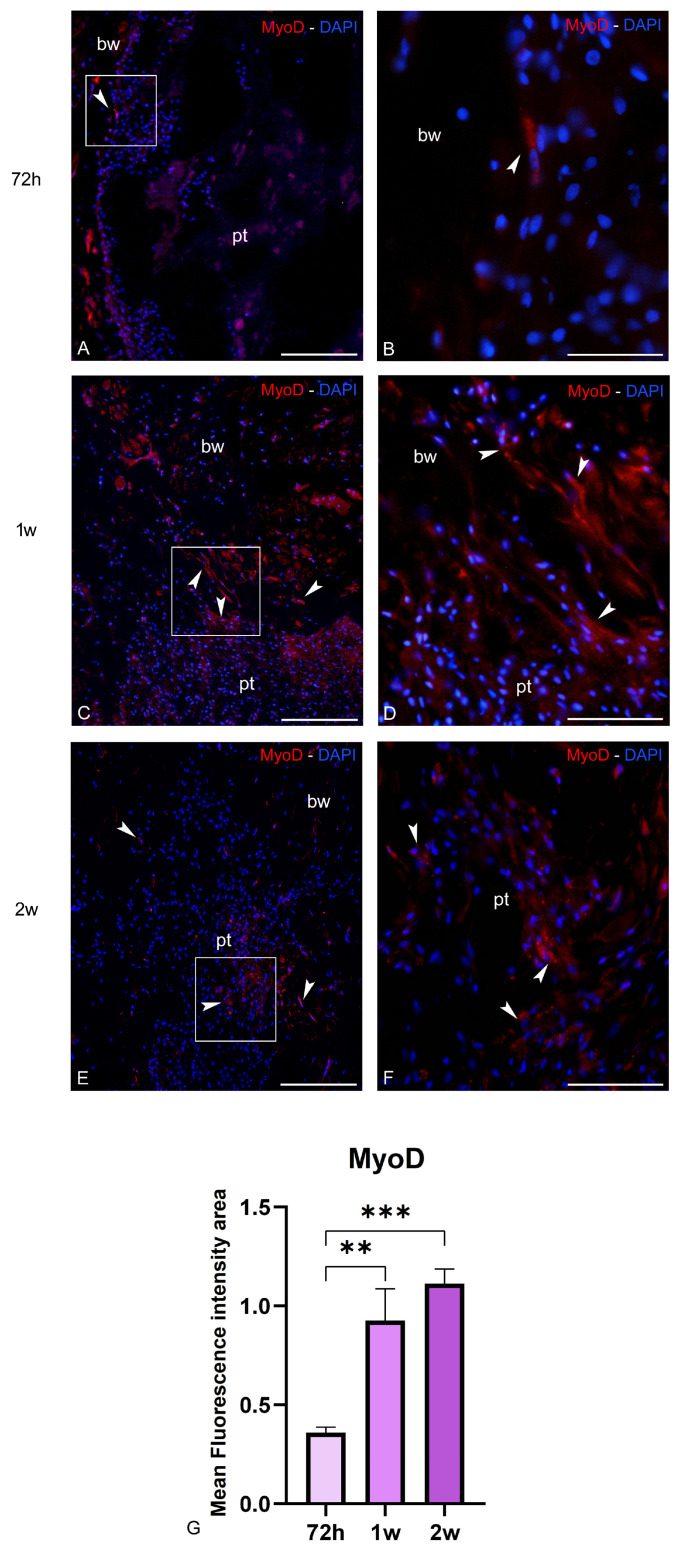
Myogenic activation and MyoD^+^ cell recruitment within the patch. (**A**–**F**) Representative immunofluorescence micrographs showing MyoD expression (red) in patch (pt) sections at 72 h, 1 week, and 2 weeks post-implantation. Nuclei are counterstained with DAPI (blue). (**A**,**B**) At 72 h, MyoD^+^ cells (white arrowheads) are rare and localized primarily near the host body wall (bw) interface. (**C**,**D**) At 1 week and (**E**,**F**) 2 weeks, a marked increase in MyoD^+^ cells (white arrowheads) is observed, with immunopositive precursors extensively infiltrating the inner regions of the graft, suggesting sustained myogenic activation during the remodeling process. (**G**) Quantitative analysis of the fluorescence intensity area confirms a significant temporal increase in the MyoD signal from 72 h to 2 weeks (*p* < 0.01. Statistical significance is indicated as *** (*p* < 0.001)). White boxes in (**A**,**C**,**E**) indicate the magnified regions shown in (**B**,**D**,**F**), respectively. Scale bars: (**A**,**C**,**E**): 100 μm; (**B**,**D**,**F**): 50 μm. The specificity of all used primary antibodies was confirmed by negative control experiments, in which the omission of the primary antibody resulted in a complete absence of signal (Figure S2).

MyoD immunostaining showed the presence of myogenic cells within and around the patch at all examined time points, with a progressive increase over time. At 72 h (Figure 6A,B), MyoD-positive cells were rare and predominantly were detected close to the tissue–patch boundary, suggesting an early phase of cellular recruitment and myogenic activation in response to implantation.

At 1 week (Figure 6C,D), MyoD immunoreactivity became more evident, with positive cells distributed both at the periphery and within the patch interior. The increased number of MyoD-positive cells suggested that the patch provided a suitable environment for the persistence and/or recruitment of myogenic progenitor-like cells during the early stages of tissue repair. In several regions, MyoD-positive cells appeared aligned within the patch architecture, compatible with active tissue organization.

At 2 weeks (Figure 6E,F), MyoD-positive cells remained detectable within the patch, although their distribution appeared more localized than at 1 week. Their persistence at this later time point indicated that myogenic activity was maintained during the remodeling phase, supporting a continued regenerative response within the implanted patch. Taken together, these data suggested that the patch favored the establishment of a myogenic microenvironment over time. Quantitative analysis of MyoD fluorescence intensity revealed a significant increase from 72 h to 1 week, with persistence of a strong signal at 2 weeks, supporting sustained myogenic commitment during patch remodeling (Figure 6G).

## 4. Discussion

The clinical management of chronic wounds remains a significant burden for healthcare systems worldwide, primarily due to the failure of the physiological healing phases to progress beyond a state of persistent, pro-inflammatory stagnation [3,4,36]. In this context, the development of advanced biomaterials capable of actively modulating the wound microenvironment is essential. In this study, we evaluated the regenerative potential of a 3D gelatin–chitosan-based patch functionalized with Mg-doped hydroxyapatite (MgHA), using the medicinal leech *Hirudo verbana* as an experimental model. Our results demonstrate that this scaffold does not merely act as a passive physical barrier; instead, it achieves rapid structural integration and actively orchestrates a complex, multi-lineage cellular response that leads to highly organized tissue reconstruction.

To better evaluate the biological impact of the GelMgHA@GelChit patch, the morphology of the treated area was compared with that of untreated wounds allowed to heal spontaneously. Morphological analysis of the control group revealed a typical physiological repair process characterized by a standard inflammatory infiltrate and less organized connective tissue deposition. In contrast, the application of the bioactive patch induced a more coordinated cellular response, with morphological changes being particularly evident at early time points (72 h). While specific immunofluorescence markers (CD31, CD34, MyoD, and FGFr) were primarily used to characterize the integration and the pro-regenerative environment within the scaffold, the comparative morphological evidence suggests that the presence of Mg-doped hydroxyapatite redirects the natural healing path toward more structured tissue remodeling from the very beginning of the process.

By promoting the organized recruitment of myoendothelial and fibroblastic cells, the patch effectively modulates the wound microenvironment. This orchestrated transition not only favors tissue reconstruction but also potentially limits the persistence of the inflammatory phase, which is often a precursor to bacterial colonization. Consequently, this bioactive strategy could indirectly mitigate the risks associated with chronic infections by promoting timely and high-quality wound closure.

### 4.1. Patch Integration and the Role of the Pseudoblastema

A crucial requirement for any effective biomaterial is its “bio-receptive” capacity—the ability to support immediate cell migration and proliferation. Our morphological and SEM analyses confirmed that the patch’s intrinsic adhesive properties and interconnected porosity facilitated near-immediate colonization by host cells. In the leech model, the earliest defensive and regenerative response to injury involves the mass migration of vasocentral cells toward the wound site to form the pseudoblastema, a transient regenerative tissue [32]. Our TEM data at 72 h provided critical evidence of this phenomenon, showing vasocentral cells lining the scaffold architecture and infiltrating its pores. This indicates that the 3D patch acts as an inductive template for pseudoblastema assembly. This initial “homing” phase is fundamental: the patch effectively “captures” these versatile precursors, establishing the necessary cellular foundation for subsequent phases of tissue maturation and graft incorporation.

### 4.2. The Myofibroblast Role in ECM Remodeling

Collagen plays a crucial role in the wound healing process by providing structural support to the tissue [37]. In leeches, the connective tissue serves as a scaffold, facilitating tissue reconstruction, new vessel formation, and cell migration and differentiation [22,23]. Masson’s Trichrome staining revealed a dynamic, time-dependent maturation of the extracellular matrix (ECM). The transition from a loose, light-blue matrix at 72 h to a dense, compact dark-blue connective tissue by the end of the second week signifies a transition from early provisional matrix deposition to a stable, mature connective tissue.

This remodeling was functionally linked to the activation of fibroblast-like cells, specifically myofibroblasts. By employing FGFr as a marker, which, in the leech, identifies the activated state of these cells [19,38], we confirmed that the synthesis of new collagen fibers is a continuous, regulated process. Since these myofibroblasts derive from the same versatile vasocentral population that forms the pseudoblastema, their sustained FGFr expression within the patch suggests that the gelatin–chitosan environment enriched with MgHA provides specific biochemical cues that maintain their biosynthetic activity.

### 4.3. Neo-Angiogenesis as a “Biological Highway” for Recruitment

A pivotal finding of our study is the role of neo-angiogenesis as a prerequisite for deep patch colonization. In leeches, healing involves the rapid rearrangement of the botryoidal tissue, an endogenous reservoir of angiogenic factors and Hematopoietic Stem and Progenitor Cells (HSPCs), into new tubular structures [20,39]. The significant peak in CD31^+^ expression observed at one week marks the peak of this vascularization phase. We interpret this vascular surge not merely as a structural event for nutrient delivery, but as a “biological highway” for systemic cellular influx. The increased vascular density likely facilitates the massive recruitment of HSPCs and myoendothelial precursors from the host’s circulation and adjacent tissues into the patch’s innermost regions. The patch, therefore, effectively “harnesses” the host’s vascular response to bridge the gap between peripheral recruitment and internal graft maturation.

### 4.4. Myogenic Commitment and Functional Tissue Integration

The final and most complex stage of the regenerative process is the differentiation of these recruited precursors into functional tissue. The co-expression of CD34 and MyoD is considered a hallmark of the myogenic program in the leech, identifying cells that have transitioned from a migratory progenitor state to a myogenic lineage [21]. Our immunofluorescence analysis revealed a clear temporal progression: while the vascular marker (CD31) stabilized by the second week, suggesting maturation of the new vessels, the expression of CD34 and MyoD remained prominent. This persistent positivity, coupled with TEM evidence showing the presence of differentiating muscle fibers characterized by organized myofilaments, confirms that the recruited myoendothelial cells successfully undergo myogenic commitment within the patch. This demonstrates that the patch–host interface is a site of active myogenesis, leading to complete functional integration of the graft into the host body wall.

A limitation of the present study is the lack of a direct assessment of Mg^2+^ release kinetics from the GelMgHA@GelChit scaffold. Although previous studies on Mg-substituted biomimetic apatites reported progressive ion-release behavior quantified by ICP-OES analyses and demonstrated the biological relevance of Mg^2+^ release for tissue regeneration, the present work was specifically focused on the in vivo evaluation of scaffold integration and regenerative performance. Future investigations will address the quantitative correlation between scaffold degradation, Mg^2+^ release kinetics, and biological outcomes.

## 5. Conclusions

Taken together, our results highlight the exceptional potential of 3D gelatin–chitosan scaffolds functionalized with Mg-doped hydroxyapatite as innovative tools for tissue engineering. The *H. verbana* model proved to be a highly predictive and efficient system, revealing an orchestrated, multi-step recruitment of vasocentral cells, HSPCs, and myoendothelial progenitors. The comparison with untreated controls demonstrated that while the leech possesses an innate healing capacity, the presence of the bioactive patch redirects this process toward more structured and organized tissue reconstruction, with significant morphological improvements already evident at the earliest stages of repair (72 h). The synergistic interplay between rapid collagen deposition by FGFr^+^ myofibroblasts and the vascular-mediated recruitment of myogenic precursors underscores the biomimetic efficiency of the patch. By providing both a physical structure and a supportive microenvironment, the patch effectively directs the endogenous regenerative potential of the organism toward successful functional integration. Furthermore, the promotion of a timely and coordinated healing process suggests that such bioactive scaffolds could play a crucial role in preventing the persistent inflammation that leads to bacterial colonization.

Future studies will investigate the immunomodulatory effects of this patch in greater detail, focusing on its influence on macrophage-like cell activity and its performance in infected wound environments. A key advantage of this platform is the potential to customize the scaffold through the incorporation of antibacterial agents or specific bioactive molecules. This research path could offer new therapeutic avenues for the treatment of refractory chronic wounds in vertebrate clinical settings, adhering to the 3Rs principle through the use of validated invertebrate models. Furthermore, the ability to personalize the patch with targeted antimicrobial or bioactive payloads could significantly enhance its regenerative efficacy and facilitate tailoring for complex clinical needs.

## Figures and Tables

**Table 1:** List of primary antibodies used for immunocytochemical studies.

Antibody	Description	Company	Dilution
CD34	Rabbit monoclonal	abcam	1:100
CD31	Mouse monoclonal	Novocastra	1:200
MyoD	Rabbit polyclonal	abcam	1:100
FGFr	Rabbit polyclonal	Santa Cruz Biotechnology	1:100

## Data Availability

The data presented in this study are available in the article and Supplementary Materials.

## Supplementary Materials

The following supporting information can be downloaded at: https://www.mdpi.com/article/10.3390/nano16120712/s1. Figure S1. Histological analysis of wound healing and tissue regeneration in *H. verbana* body wall. (A–C) Representative images of cross sections stained with Masson’s Tricrome Staining at different time points after experimental injury: (A) 72 h (72 h), (B) 1 week (1 w), and (C) 2 weeks (2 w) post-lesion. The blue staining highlights the deposition of collagen fibers and extracellular matrix (ECM) components, while muscle fibers and cells are stained in red/orange. Scale bars: 200 µm. Figure S2. Immunofluorescence negative controls and marker localization in the injured area. (A–D) Representative fluorescence micrographs showing the distribution of nuclei (DAPI, blue) and the absence of non-specific signals for key regenerative and vascular markers: (A) FGFr, (B) CD34, (C) CD31, and (D) MyoD. The images confirm the specificity of the primary antibodies used to track the recruitment of precursor cells and the activation of the neo-angiogenetic process in the perilesional tissue (pt) and body wall (bw) adjacent to the wound site. Scale bars: 100 µm.
