# Supplementary material for: Evaluating 3D-Patch Efficacy in Wound Healing Using the Medicinal Leech Hirudo verbana as an In Vivo Model
Source: Nanomaterials (Basel). 2026 Jun 9;16(12):712. doi: 10.3390/nano16120712 (PMC13305015; doi:10.3390/nano16120712)
Supplement: Supplementary file 1 [file nanomaterials-16-00712-s001.zip › Figure legends supplemetry figures.pdf]

Figure legends supplemetry figures

Figure S1. Histological analysis of wound healing and tissue regeneration in *H. verbana* body wall.
(A–C) Representative images of cross sections stained with Masson's Tricrome Staining at different time points after experimental injury: (A) 72 hours (72h), (B) 1 week (1w), and (C) 2 weeks (2w) post-lesion. The blue staining highlights the deposition of collagen fibers and extracellular matrix (ECM) components, while muscle fibers and cells are stained in red/orange. Scale bars: 200 µm.

Figure S2. Immunofluorescence negative controls and marker localization in the injured area. (A–D) Representative fluorescence micrographs showing the distribution of nuclei (DAPI, blue) and the absence of non-specific signals for key regenerative and vascular markers: (A) FGFr, (B) CD34, (C) CD31, and (D) MyoD. The images confirm the specificity of the primary antibodies used to track the recruitment of precursor cells and the activation of the neo-angiogenetic process in the perilesional tissue (pt) and body wall (bw) adjacent to the wound site. Scale bars: 100 µm.
