# Supplementary figures and images for: Evaluating 3D-Patch Efficacy in Wound Healing Using the Medicinal Leech Hirudo verbana as an In Vivo Model
Source: Nanomaterials (Basel). 2026 Jun 9;16(12):712. doi: 10.3390/nano16120712 (PMC13305015; doi:10.3390/nano16120712)

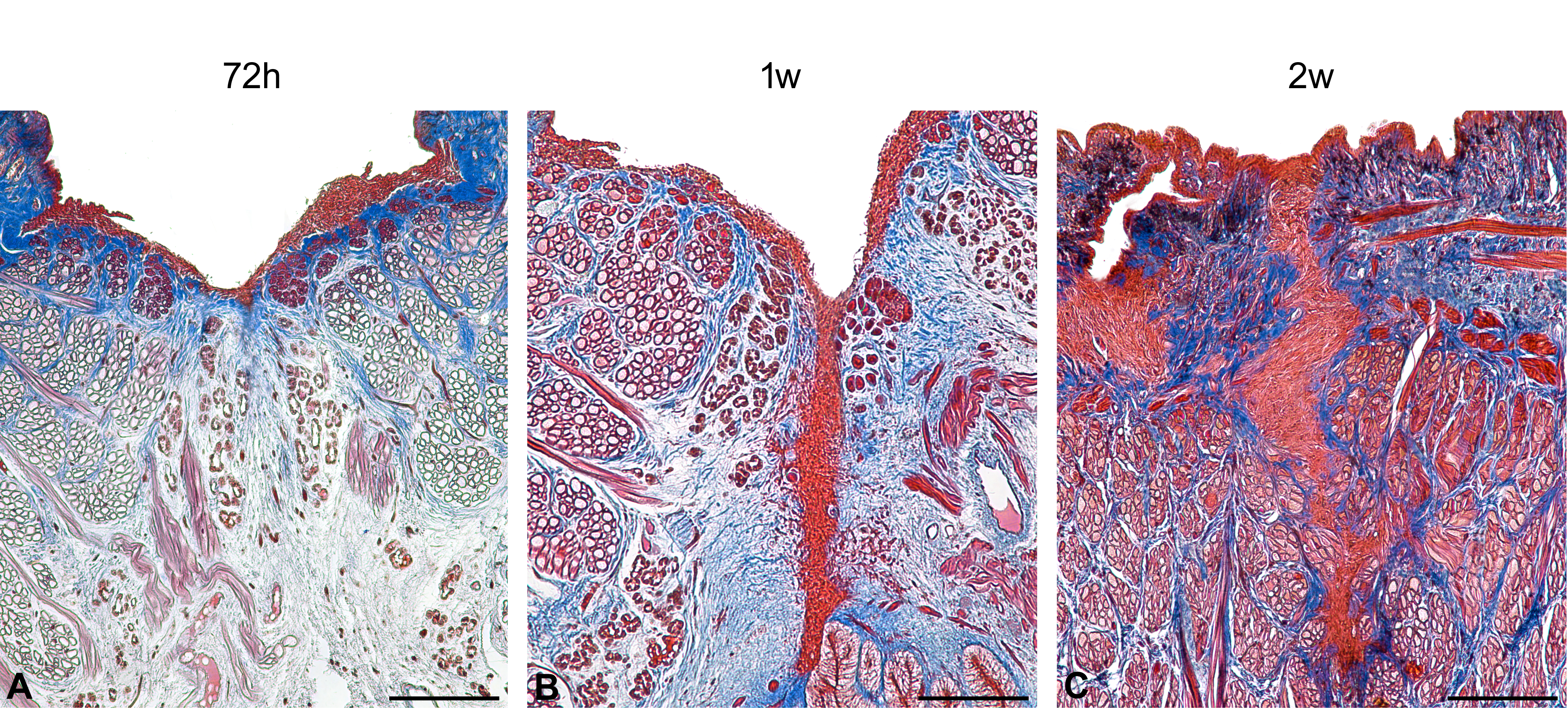

Supplement: Supplementary file 1 [file nanomaterials-16-00712-s001.zip › Fig S1.tif]

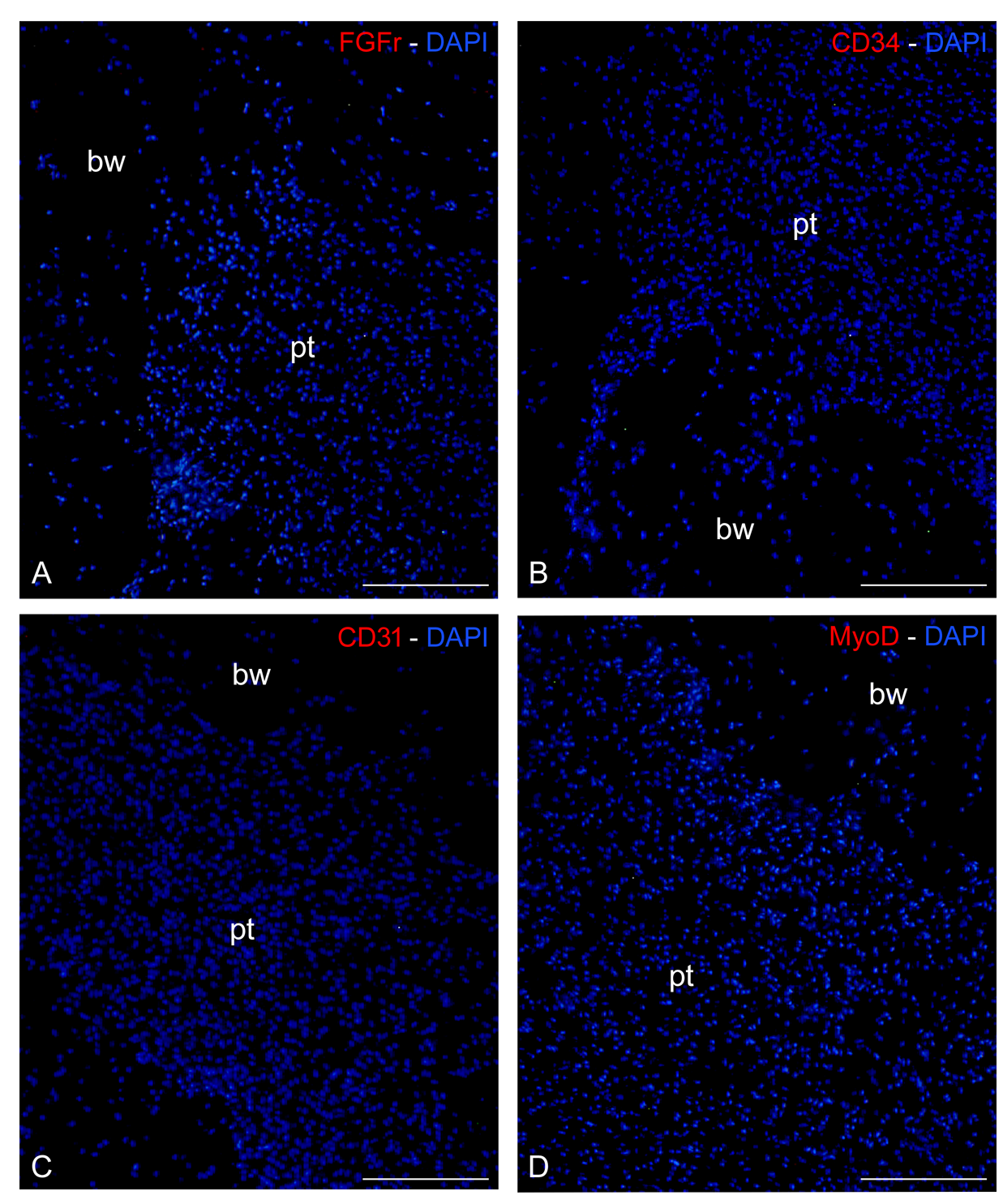

Supplement: Supplementary file 1 [file nanomaterials-16-00712-s001.zip › FigS2.tif]
